# 

**Published:** 1990-06

**Authors:** 


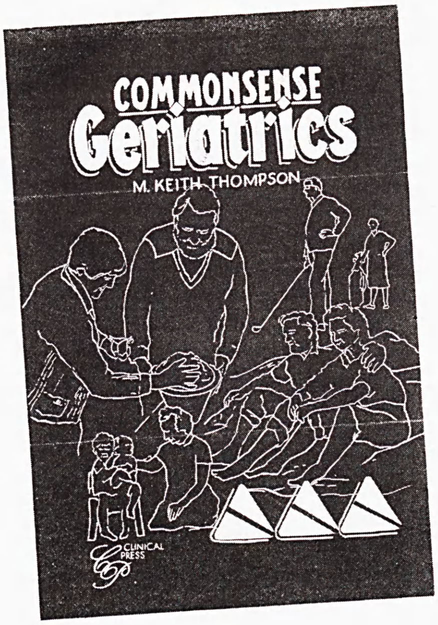

### Commonsense GERIATRICS

Keith Thompson, General Practitioner, London

Examiner for the Diploma in Geriatric Medicine

Mankind is ageing. For the first time in history the elderly make up a steadily increasing proportion of the population in many countries. In the face of this unprecedented change in the age structure of society the teaching and practice of geriatric medicine is patchy, much of it concerned with rehabilitation.

**Commonsense Geriatrics** sets out to provide a commonsense, practical approach, both clinical and organisational, to geriatric care for the GP and the specialist. This volume is compact, clear and interesting and it is written with the aim of making a substantial branch of medical and social practice manageable by a structured approach.

The author, is an eminent GP who can draw on his experience of medical services all over the world. Dr. Thompson has an international reputation as a writer, teacher, broadcaster and researcher all based on long experience in general practice. The **Diploma in Geriatric Medicine** is now recognised as an important postgraduate qualification and this volume will be of value to candidates. The author is an examiner for the Diploma.

CONTENTS: First principles—Population and population structure—Old people in the practice—Basic principles—Attitudes in the care of the elderly—Special difficulties with the older patients—Practice organisation—The team concept—The examination—The investigation—Health education and follow up—The ageing process—Common clinical problems—Speciality aspects—The whole person—Index.

150 pages. Papercover. Fully illustrated. ISBN 1-85457-007-2. £12.50.

**ORDER FORM**

Please order from your usual bookseller or direct from Clinical Press Ltd., c/o Gazelle Book Services, Falcon House, Queen Square, Lancaster, LA1 1RN, UK

Please send me

............. copy(s) of ..........................................................................................

............. copy(s) of ..........................................................................................

............. copy(s) of ..........................................................................................

**Prepaid orders or credit-card orders will be supplied post free**

I enclose a cheque for ....................... (Payable to Gazelle Book Services)

OR
I authorise you to charge my Access/Barclaycard/American Express credit account

Card: ............................................... Number: ............................................

Expiry Date: ..................................... Signature: ..........................................

OR
Please invoice me at list price plus £1.50 per copy postage and packing

Name: (Please Print) ..........................................................................................

Address: (Please Print) ......................................................................................

For those suffering from incontinence on exertion or following pelvic surgery.

### REGAINING BLADDER CONTROL

*Second Edition*

Eileen Montgomery, MCSP

Far too many people suffer from unintentional leakage of urine.

Eileen Montgomery provides a positive approach to the control of this problem focusing directly on the weakness of the pelvic muscles.

This important patient handbook contains a planned series of exercises aimed at restoring control of the bladder. The anatomical causes of incontinence are clearly explained and illustrated. The book also deals with correct breathing and a balanced diet which will lead to constipation or to being overweight.

Surgeons, doctors and physiotherapists will be able to recommend this booklet to their patients.

28 pages. Soft cover. 11 line drawings. ISBN: 1-85457-003 X. Price £2.50 per copy or £20.00 per pack of 10 copies.

**Editorial Committee**
Chairman of Editorial Board, Professor John Farndon
Editor—Mr M. G. Wilson
Assistant Editors—Dr Paul Goddard, Dr Alison Round
Secretary—Mr Clive Ward
Members—Dr John Burton, Dr Kenneth Southgate

**Editorial Office**—*The Red House, 40 Coombe Lane, W.O.T., Bristol BS9 2BJ*
*Tel 0272 682320*

**Correspondents**
Dr Andrew Adam (Taunton)
Mr Martin Crosfil (Truro)
Dr Jack Davies (Bristol)
Professor Denis Pereira Gray (Exeter)
Dr Jose Jancar (Bristol)
Dr Michael Oliver (Barnstaple)
Professor Gordon Stirratt (Bristol)
Dr Michael Whitfield (Bristol)
Professor Gordon Wilcock (Bristol)

**Associated Societies**
Bristol Medico-Chirurgical Society
Clinical Society of Bath
Severn Faculty of the Royal College of General Practitioners
South West of England and Wales Society of Dermatology
South West Obstetricians and Gynaecologists Club
South West Orthopoedic Club
South West Paediatric Club
South West Radiologists Club
Surgical Club of South West England
Tamar Faculty of the Royal College of General Practitioners
Wessex Association of Clinical Pathologists
West Country Chest Physicians Club
West Country Physicians Club

Members of the above organisations receive the journal as part of their membership subscription.
Annual Subscription Rate £25.00 including surface postage.

**Published by**
Clinical Press
*15 Bucklands Drive, Nailsea, Bristol BS19 2PH*

**Typesetting by**
*Apek Typesetting*
*Avon House, Nailsea, Bristol BS19 2DJ*

**Printed by**
*Sutton Print, Paignton*

**THE WEST OF ENGLAND MEDICAL JOURNAL acknowledges with gratitude the help it receives from NUFFIELD HOSPITALS**

### WEST OF ENGLAND MEDICAL JOURNAL

1883–1989 Vols 1–104

**NOTICE TO CONTRIBUTORS**
Contributions are invited especially from West of England authors on a variety of subjects, e.g. Original articles relating to the practice of medicine, reports on the contemporary medical scene, obituaries, historical accounts, recollections of colleagues and events worthy to be remembered and liable to be forgotten. An article of 2,000 words with 2 illustrations will occupy 2 pages and this is a suggested, though by no means an obligatory length. Articles are preferred in the form proposed by the International Committee of Medical Editors (Vancouver System)—pamphlet obtainable from Editor of BMJ., BMA. House, Tavistock Square, London, WC1H 9JR. Please send two copies retaining a further copy for your reference.

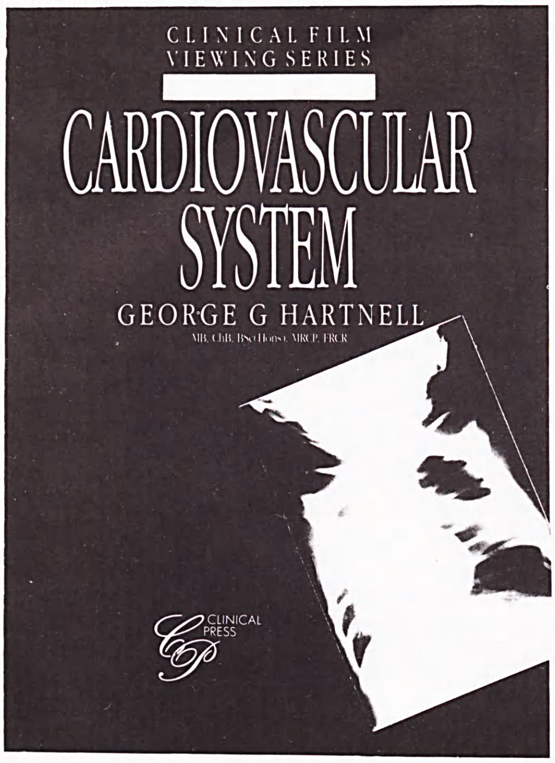

**Self assessment radiography**

### Clinical Film Viewing Series

Series Editor: Paul R. Goddard, MD., FRCR

Most postgraduate examinations now include radiographic interpretation. This may be presented as a slide show or as a film-viewing session. Radiographs and other imaging modalities are also used including those of the American Boards and professional societies and universities world wide.

In a typical examination situation, candidates are asked to view a film but are provided with minimal clinical information. They are then asked to give a written or oral report together with a sensible list of possible differential diagnoses.

This series of books on *Clinical Film Viewing* will assist candidates to prepare for this process. Each book examines a particular system or technique and promotes the reader's self-questioning about the film presented. The series provides a unique opportunity for self-assessment and learning in all the modern modalities of diagnostic imaging.

Each book consists of an introduction to the speciality or technique followed by eighty profusely illustrated cases typical of those presented in postgraduate examinations.

**Now available**

### CARDIOVASCULAR SYSTEM

George G. Hartnell, MB., ChB., BSc(Hons)., MRCP., FRCR.

'An invaluable learning book' from the Foreword by Professor E.R. Davies, *Department of Radiodiagnosis, Bristol Royal Infirmary.*

180 pages. 216mm x 138mm. Papercover. Over 100 illustrations. ISBN: 1-85457-0123-9. Price £15.00

**To be published soon**

### RESPIRATORY SYSTEM

Paul R. Goddard, BSc., MB BS, MD, DMRD, FRCR

200 pages 216mm x 138mm. Papercover. Over 110 illustrations. ISBN 1 85457 014 5

**Titles in the Series**

Moshy – General Introduction

Hartnell – Cardiovascular System

Goddard – Respiratory System

Kabala and Slack – Ultrasound

Lewis – Central Nervous System

Watt – Muskuloskeletal System

Virjee – Gasterointestinal System

Henson – Obstetrics and Gynaecology

Birch – Accident and Emergency

Banerjee – AIDS

Cook – Computed Tomography

Cook – Magnetic Resonance Imaging

---

**A concise guide for radiologists and radiographers.**

### LECTURE NOTES ON THE PHYSICS OF RADIOLOGY

Susan J. Armstrong, MB., ChB., MRCP., Registrar, Department of Radiodiagnosis, Bristol Royal Infirmary

'Susan Armstrong's book will be a companion throughout the course and a good read during the last anxious days before the exam. This is a book which will serve as a handy, concise and comprehensive reference in the years ahead. Written in an attractive style and free from superfluous verbiage, it will be immensely valuable to radiologists, radiographers, physicists, technicians and other professionally involved in radiology.' *From the Foreword by Professor Peter Wells, Honorary Professor of Radiodiagnosis, Bristol Royal Infirmary. Lecture Notes on the Physics of Radiology* is suitable for study for the higher qualifications in radiology and radiography.

CONTENTS: Atomic Structure and Radioactivity—The Production of X-Rays—X-Ray Generating Apparatus—Interactions between X-Rays and Matter—Dosimetry—Films and Screens—Image Quality—Fluoroscopy—Special Techniques—Computed Tomography—Ultrasound—Radionuclide Imaging—Magnetic Resonance Imaging—Quality Control—Radiation Protection—Bibliography—Index.

216 pages. Papercover. 216mm × 148mm. 98 illustrations. ISBN: 1-85457-010 2. £15.00.

Please order from your usual bookseller or direct from Clinical Press Ltd., c/o Gazelle Book Services, FalconHouse, Queen Square, Lancaster, LA1 1RN, UK.

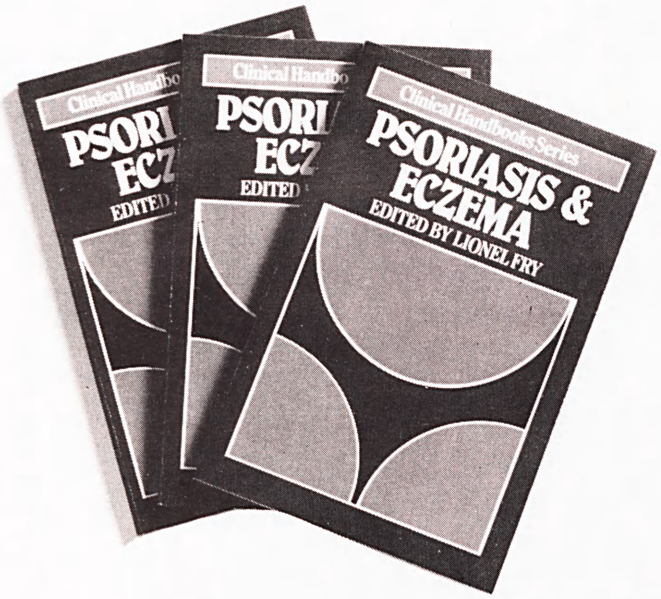

### Clinical Handbooks Series

This series is intended particularly for young doctors working in hospitals and primary care and for those training them. The series has been designed to cover growing points in medicine and the authors have been chosen, not just because of their expertise, but also because they are working both in hospitals and the community and are thus sensitive to the problems and needs of doctors in both areas.

**HEART FAILURE** Edited by Gerald Sandler, Consultant Physician, Barnsley

Contents: Pathophysiology of Heart Failure. Treatment of Heart Failure. The Place of Surgery in the Management of Common Heart Conditions. The Clinical Aspects of Heart Failure.

100 pages. Papercover. ISBN 1 85457 008 0. £10.00 approx.

*The first title in an important new sieries*

### PSORIASIS AND ECZEMA

*Edited by Dr. Lionel Fry, Consultant Dermatologist, St. Mary's Hospital, London.*

The book is intended to give some insight into recent advances in the pathogenicity of these diseases as well as their clinical features and management.

*Contributors:* Psoriasis: Clinical Features (L. Fry) – The Treatment of Psoriasis (C.E.M. Griffiths) – The Aetiology and Pathogenesis of Psoriasis (B.S. Baker) – Eczema: Clinical Features (R.J. Clayton) – The Manasgement of Exzema (J.N. Leonard) – The Aetiology of Eczma (A.V. Powles) – Index.

120 pages. Paper cover. Fully illustrated in colour.

ISBN 1 85457 0002 1. £15.00

**SECOND OPINIONS IN INTERNAL MEDICINE** Edited by John Fry, General Practitioner, London, Gerald Sandler, Consultant Physician, Barnsley *and* Wesley Fabb, General Practitioner, Melbourne.

The problem of time wasting, unnecessary referral to specialist by primary care practitioners is universal. This unique book provides young doctors with a series of cases through which they can develop their judgment of whether to refer or not.

Contents: Jaundice – Resistant hypertension – Breathlessness – Dyspepsia – 'Mrs Neverwell' – Headaches – Swollen legs – Polyarthritis – Chest pain – 'Funny turns' – Dizziness – Backpain – Index

150 pages. Papercover. ISBN 1 85457 019 6. £15.00 approx.

### Available soon

**DIABETES** Edited by Arun Baksi, Consultant Physician, St. Marys Hospital, Newport, IoW

Contents: What is diabetes mellitus – Diagnosis and calssification – Objectives of diabetes therapy – Medical treatment of non-insulin-dependent diabetes – Insulin treatment – The complications of diabetes – Index

120 pages. Papercover. Fully illustrated. ISBN 1 85457 005 6. £12.50

**ORDER FORM**

Please order from your usual bookseller or direct from Clinical Press Ltd., c/o Gazelle Book Services, Falcon House, Queen Square, Lancaster, LA1 1RN, UK

Please send me

................ copy(s) of ..........................................................................

................ copy(s) of ..........................................................................

................ copy(s) of ..........................................................................

**Prepaid orders or credit-card orders will be supplied post free**

I enclose a cheque for .......................... (Payable to Gazelle Book Services)

OR
I authorise you to charge my Access/Barclaycard/American Express credit account

Card: ................................................ Number: ......................................

Expiry Date: ...................................... Signature: ....................................

OR
Please invoice me at list price plus £1.50 per copy postage and packing

Name: (Please Print) ..........................................................................

Address: (Please Print) ......................................................................

